# Anti-C2–mediated complement inhibition prevents disease in a rat passive transfer model of myasthenia gravis

**DOI:** 10.1016/j.jtauto.2026.100388

**Published:** 2026-07-16

**Authors:** Britt Arets, Siyang Peng, Laura Bracke, Karen De Winter, Jolien Delaere, Yasmine Driege, Tim Delahaye, Mahan Moshir, Inge Van de Walle, Peter Ulrichts, Marina Mané-Damas, Mario Losen, Pilar Martinez-Martinez

**Affiliations:** a Department of Psychiatry and Neuropsychology, Mental Health and Neuroscience Research Institute, Maastricht University, the Netherlands; b Department of Translational Sciences, argenx, Ghent, Belgium

**Keywords:** Myasthenia gravis, Passive transfer, Complement blockade, Complement C2

## Abstract

Acetylcholine receptor antibody-positive myasthenia gravis (AChR-MG) is a rare autoimmune disease in which AChR autoantibodies trigger complement activation and cause damage to the neuromuscular junction. This study evaluated the therapeutic potential of an anti-C2 antibody in the rat passive transfer model of myasthenia gravis (PTMG). Female Lewis rats (n = 36) were prophylactically treated with either an anti-rat C2 antibody or an IgG isotype control 2 h prior to subcutaneous injection of mAb35 (PTMG) or saline (non-MG). Serum samples were collected at several timepoints during the study and muscle weakness; body weight and clinical manifestations were assessed daily. The animals underwent electromyography prior to euthanasia. AChR content in the tibialis anterior was determined by radioimmunoassay. Anti-C2 antibody-treated animals maintained body weight, grip strength and muscle AChR content (both functional and total) at levels comparable to non-MG animals. Serum analyses confirmed reduced complement activity over the course of the experiment in anti-C2 treated animals. These findings indicate that anti-C2 treatment effectively prevents disease development in this model, supporting its potential as a therapeutic strategy for managing AChR-MG.

## Abbreviations

AChR –: Acetylcholine receptor
AChR-MG –: Acetylcholine receptor antibody-positive myasthenia gravis
BW –: Body weight
C2 –: Complement component 2
C3 –: Complement component 3
C5 –: Complement component 5
CP –: Classical pathway
EMG –: Electromyography
LP –: Lectin pathway
MAC –: Membrane attack complex
mAb35 –: Monoclonal antibody 35
NMJ –: Neuromuscular junction
PTMG –: Passive transfer model of myasthenia gravis
TA –: Tibialis anterior
TCC –: Terminal complement complex

## 1 Introduction

Beyond its established role in host defense, complement activation is a critical driver of tissue injury and disease in many antibody-mediated autoimmune diseases, including myasthenia gravis, neuromyelitis optica spectrum disorder, autoimmune hemolytic anemia, systemic lupus erythematosus, antineutrophil cytoplasmic antibody (ANCA)‐associated vasculitis and antiphospholipid syndrome [1]. Therefore, inhibition of the complement cascade has emerged as a promising therapeutic strategy in these diseases. The complement system comprises more than 30 soluble and membrane-bound proteins activated through the classical, lectin, and alternative pathways, which converge at C3 to form the membrane attack complex (MAC) [2]. Cell-bound IgM, densely clustered IgG1 and IgG3 autoantibodies, and immune complexes containing these antibodies can initiate the classical complement pathway through C1 complex binding. Several complement inhibitors are clinically approved, including C5 blockade and C1 inhibitors, but none are universally effective [2,3]. C5 inhibition does not prevent C3-mediated inflammation, and C1 inhibitors have limited efficacy against antibody–bound C1 [2]. As a result, additional complement targets are being explored for therapy.

Complement component 2 (C2) occupies a key position in both the classical and lectin pathways and therefore represents an attractive, but currently untapped target. Recently, empasiprubart, a high-affinity monoclonal antibody targeting C2 has been developed to inhibit formation of the C4bC2 complex and thus subsequent complement activation [2,4]. This approach has demonstrated safety in phase 1 and clinical efficacy in phase 2 studies in patients with multifocal motor neuropathy [5]. Given the central role of classical pathway complement activation in acetylcholine receptor antibody-positive myasthenia gravis (AChR-MG), targeting C2 represents a promising repositioning strategy.

AChR-MG is characterized by chronic, fatigable skeletal muscle weakness due to AChR loss at the neuromuscular junction (NMJ). AChR antibodies are predominantly of the IgG1 and IgG3 isotypes [6] and MAC is universally present at the NMJ in muscle biopsies of AChR-MG patients [7]. Currently approved complement protein component 5 (C5) targeting drugs in use for AChR-MG such as eculizumab, ravulizumab and zilucoplan prevent MAC formation, however, they do not inhibit C3b deposition, which can still promote immune cell activation [[8], [9], [10], [11]]. In a chronic mouse model of MG, it has been demonstrated that silencing C2 mRNA using siRNA effectively reduced disease severity [12], however currently no anti-C2 drugs have entered clinical trials for MG. Other investigational drugs targeting more upstream of C2 such as C1q, C1s and MASP-2, are in development for complement mediated diseases [[13], [14], [15], [16]].

The standardized passive transfer MG (PTMG) rat model is characterized by a rapid symptom development, mimicking the acute phase of the human disease [17]. Within 48 h after injection of mAb35, a monoclonal rat IgG1 antibody against the muscle nicotinic AChR, symptoms of muscle weakness, such as body-weight loss, reduced mobility and fatigue become apparent. Biochemically, the model shows AChR loss at the NMJ and complement activation as evidenced by C3 and MAC deposition [[18], [19], [20]]. This rapid and consistent development of disease makes it a suitable preclinical model to evaluate complement inhibition therapies [[19], [20], [21], [22], [23], [24]].

A high-affinity anti-rat C2 antibody was recently developed to enable investigation of the therapeutic potential of classical and lectin pathway complement inhibition [4]. This antibody binds to the C2a part of C2, thereby blocking the formation of the C4bC2 complex and hence the activation of the rat classical and lectin pathway activity *in vitro*. Pharmacokinetic and pharmacodynamic modeling confirmed that a 50 mg/kg loading dose would maintain a reduction in free C2 concentrations ≥95% for a minimum duration of 48 h in rats. In this study we examined the ability of this rat anti-C2 antibody to prevent the development of MG symptoms in the PTMG rat model.

## 2 Results

### 2.1 Anti-rat C2 reduces classical complement pathway activity, free C2 and soluble MAC

To investigate the effect of the anti-C2 monoclonal antibody on the complement activation, free C2 levels, classical complement pathway (CP) activity (C3 deposition) and soluble MAC (sMAC) levels were analyzed in serum at baseline, 24 and 48 h post injection. In anti-C2 treated animals, free C2 levels were strongly reduced (Fig. 1A). However, an incomplete free C2 suppression (< 95.48% reduction) was observed in 5 out of 12 PTMG anti-C2 treated animals at 24 h. These 5 animals had a higher baseline body weight (BW) than the group average, suggesting that body composition such as fat distribution may have influenced the antibody’s bioavailability. At 48 h, the anti-C2 nearly completely reduced free C2 levels in all animals. In anti-C2-treated rats, the CP was inactivated compared to rats treated with an isotype control (IC) (Fig. 1B). A significant increase of sMAC levels was observed in PTMG IC animals compared with non-PTMG IC animals (Fig 1C). In both PTMG anti-C2 and non-MG anti-C2 groups, sMAC levels were significantly reduced relative to their corresponding IC controls (PTMG IC and non-MG IC, respectively), confirming systemic inhibition of the terminal complement pathway (Fig. 1C).

**Fig. 1:**
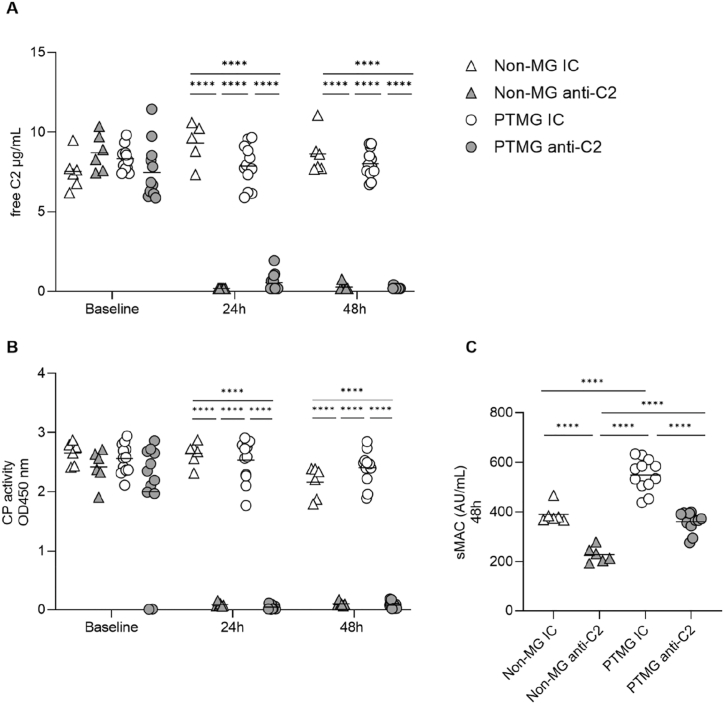
**Serum-based complement activity assessment by ELISA.** Complement activity was determined by A) free C2 at baseline, 24 and 48 h, B) classical complement pathway (CP) activity (C3 deposition) and C) soluble MAC (sMAC) at 48 h ****P ≤ 0.0001.

### 2.2 Targeting C2 prevents body weight loss and clinical symptoms of MG

Body weight was monitored throughout the study as indicator of overall health and MG disease progression. All groups-maintained BW up to 24 h post-disease induction (Fig. 2A, B). Rats treated with an IC started to lose weight between 24 and 48 h after induction of PTMG, while anti-C2 treated PTMG animals and non-MG animals maintained stable BW (Fig. 2A). At endpoint, PTMG IC animals lost 7.5% ± 2.2 of BW (Fig. 2C). The observed BW loss in PTMG IC was accompanied by progressing MG disease symptoms over the course of the study, whilst PTMG anti-C2 treated animals remained symptom-free (Fig. 2D, E).

**Fig. 2:**
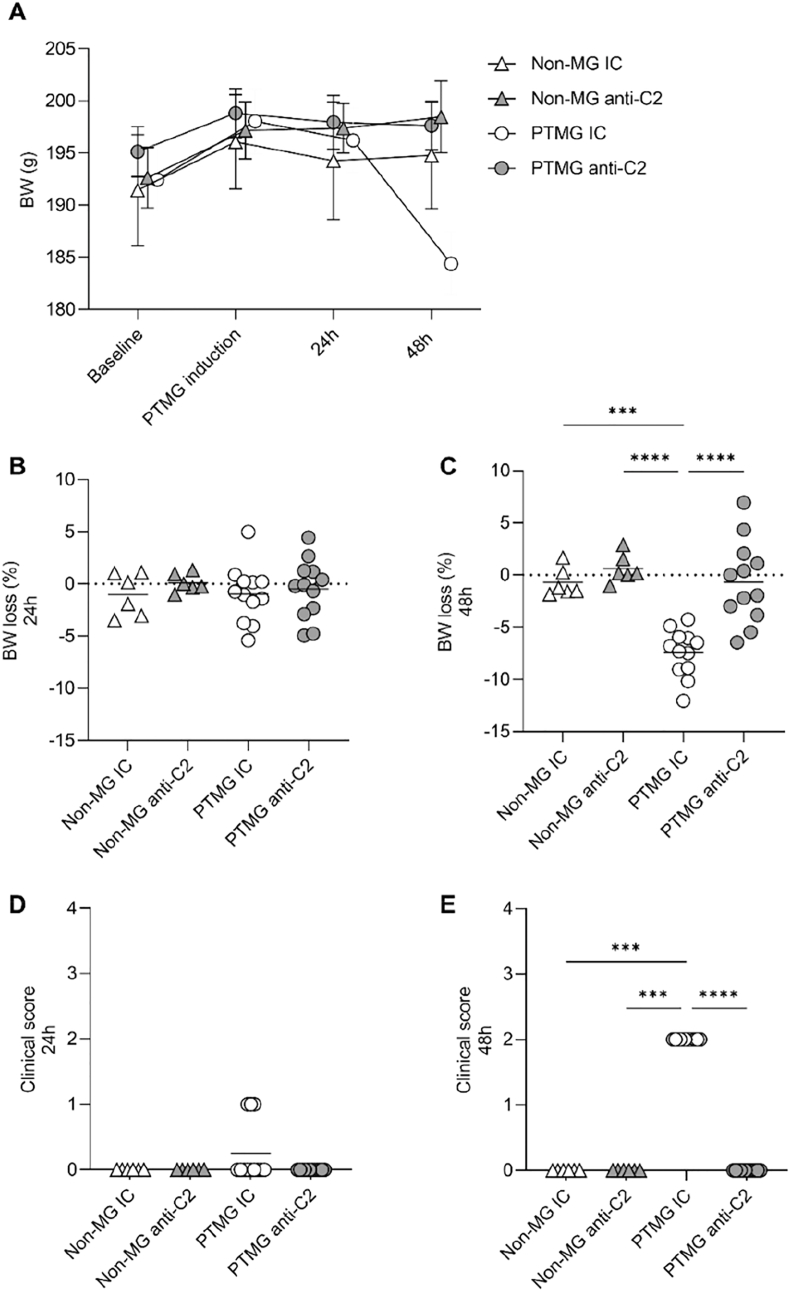
**Progression of body weight loss and clinical presentation**. A) Overview of mean body weight (BW) for each treatment group throughout the experiment. Error bars represent standard error of the mean (SEM). Percentage of BW loss at B) 24 and C) 48 h post-disease induction compared to BW at disease induction. Disease severity assessed at D) 24 and E) 48 h after disease (PTMG) induction using MG scoring. ***P ≤ 0.001, ****P ≤ 0.0001.

To assess the development of muscle weakness and fatigue, the forelimb grip-strength test was performed. PTMG anti-C2 treated animals maintained strength similar to non-MG animals, whereas PTMG IC treated animals demonstrated progressive decline of strength, with significant weakness 24 h after disease induction (Fig 3A, C). At the endpoint, the average forelimb strength in PTMG IC animals was reduced to 53% ± 16.3 relative to baseline (Supplementary Fig. S1), whereas non-MG and PTMG anti-C2 treated animals showed no significant changes (B, C). Following the grip-strength test, fatigue and overall body strength were measured using the hand climbing test. PTMG IC animals were unable to complete the task at 48 h (P<0.0001). All PTMG anti-C2 treated animals, as well as non-MG animals, were able to complete this task successfully at all time points (Fig. 3D).

**Fig. 3:**
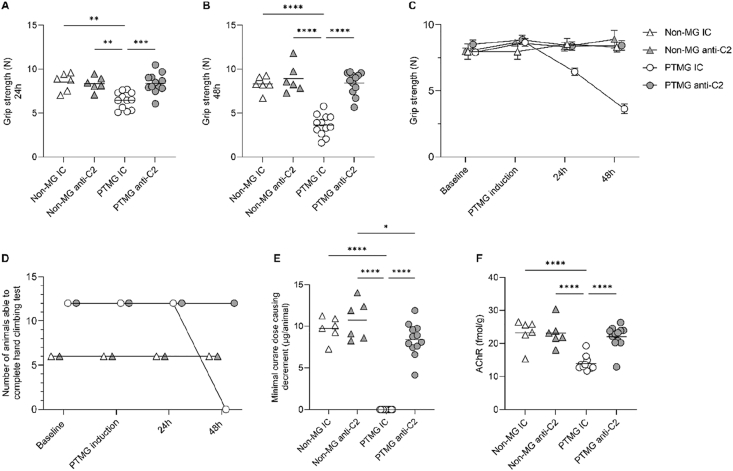
**Assessment of muscle weakness, functional AChR levels and AChR concentration in the tibialis anterior (TA) muscle.** Forearm grip strength was evaluated at A) 24, B) 48 h after PTMG induction and C) mean rack grabbing force per treatment group at all time points from baseline. Each symbol represents the average of five measurements. Errors bars represent SEM. D) Number of animals successfully performing the hand climbing task at different time points. E) The intraperitoneally infused minimal decrement-inducing curare dose during EMG. The amount of curare was determined by infusion speed and total time to achieve minimally 10% decrement. The curare dose is indicative of the number of functional AChR in the endplates. F) AChR concentration extracted from the TA. *P ≤ 0.05, **P ≤ 0.01 ***P ≤ 0.001, ****P ≤ 0.0001.

During terminal experiments, functional AChR levels were assessed by electromyography (EMG), using repetitive nerve stimulation and measurements of compound muscle action potentials (CMAPs). In all PTMG IC animals a decremental response of CMAPs (decrement) was observed, indicating that levels of functional AChRs were insufficient to allow robust neuromuscular transmission. In contrast, no decrement was observed in any of the PTMG anti-C2 treated rats. To improve the sensitivity of the decrement test and reveal subclinical NMJ dysfunction, we challenged the neuromuscular transmission systemically with a continuous infusion of curare, a competitive antagonist of nicotinic muscle AChRs. The curare dose required to elicit decrement is a measure of spare functional AChR levels at the NMJ (i.e. the safety factor), with higher doses indicating higher receptor levels [25]. In non-MG animals a mean of 10.2 μg ± 1.9 curare (Fig. 3E) was required to induce decrement. Compared to non-MG rats, curare resistance was only slightly reduced in PTMG anti-C2 treated animals with a mean decrement-inducing curare dose of 8.4 μg ± 2.0, indicating effective functional AChR preservation. Quantification of total membrane AChR in the tibialis anterior (TA) muscle confirmed preservation of the receptor in anti-C2 PTMG treated animals (Fig. 3F). With a mean receptor concentration of 13.9 fmol/g ± 2.0, PTMG IC animals lost 60.4% of their total muscle membrane AChR, whereas AChR-loss was prevented in anti-C2 treated PTMG treated animals. The mean receptor concentration in anti-C2 PTMG treated animals (22 fmol/g ± 3.4) was similar to non-MG anti-C2 animals (23 fmol/g ± 4.2).

### 2.3 Targeting C2 prevents MAC deposition at the neuromuscular junction

Next, we assessed local complement activation on NMJs by immunofluorescence. Longitudinal cryosections of tibialis anterior (TA) muscles were stained for AChR, MAC and the presynaptic synaptic vesicle marker SV2, as a reference. A qualitative assessment was performed across the entire muscle section. Compared with non-MG animals, PTMG IC animals displayed reduced AChR staining intensity together with clear MAC deposition at the NMJ (Fig. 4A, B). In contrast, anti-C2 treated PTMG animals showed AChR staining intensities comparable to non-MG anti-C2 animals (Fig. 4B). In PTMG IC rats, MAC deposition consistently co-localized with AChRs while in PTMG anti-C2 treated rats MAC staining was weak or comparable to background.

**Fig. 4:**
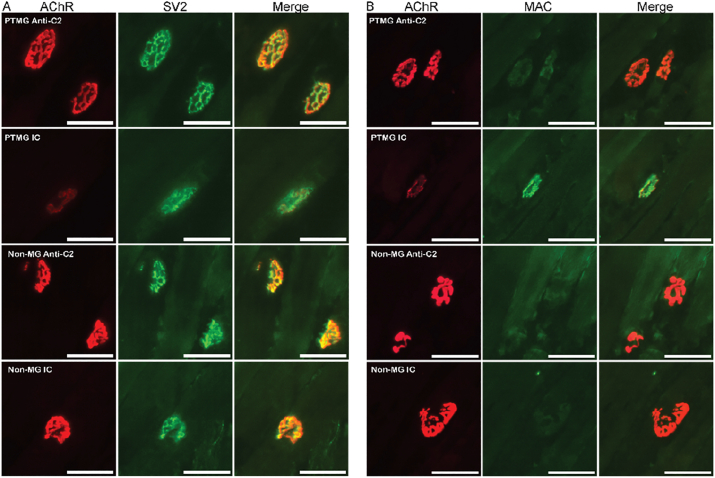
**AChR, SV2 and MAC levels in tibialis anterior (TA) muscles.** Cryosections were double stained with Alexa647-conjugated α-bungarotoxin for AChR (red) and either A) mouse anti-SV2 (green) or B) mouse anti-C5b-9 for MAC (green); merge on the right. Images depict representative NMJs; qualitative assessment was performed across the entire TA muscle. Scale bars are 50 μm.

## 3 Discussion

Complement inhibition is an emerging and increasingly important treatment approach for AChR-MG, targeting immune-mediated damage at the NMJ. Most current approaches focus on terminal pathway blockade at C5 (e.g. eculizumab, ravulizumab, zilucoplan), but targeting upstream components provides an alternative and potentially more specific means to blocking the classical and lectin pathways of complement-mediated damage. Previously, we demonstrated that silencing hepatic C3 expression ameliorated disease symptoms in the PTMG rat model [20]. In this study, we now provide proof of principle for C2 targeting in this model. Compared with C3 inhibition, targeting C2 offers a more selective approach, preserving alternative pathway-mediated immune defense while enabling combination with other immunotherapies such as B cell targeting drugs or FcRn antagonists, particularly in refractory cases or during exacerbations [26]. Upon treatment with anti-rat C2 antibody, PTMG animals were protected against disease progression, AChR loss and classical complement activation, while sMAC was only partially reduced, in line with maintained alternative pathway activity. Therefore, interfering at the C2 level might be a promising therapeutic approach for AChR-MG, either as monotherapy or in combination with other immunotherapies.

Complement analyses after anti-C2 treatment showed strongly reduced MAC deposition at the NMJ and total reduction of classical complement activity, but only partial reduction of sMAC at 48 h. The levels of sMAC were comparable to the level of non-MG rats treated with an IC antibody. As expected, similar to other classical complement pathway-based therapies, complete suppression of sMAC is not feasible as complement ‘tick over’ maintains low-level activation of the alternative pathway contributing to basal sMAC levels [10].

A previous study with the anti-C2 antibody showed complete reduction for a minimum of 48 h with a single dose [4]. We postulate therefore that the incomplete reduction of free C2 in several animals 24 h post-disease induction, could be due to intraperitoneal (I.P.) administration of the drug as body composition directly affects the pharmacokinetics of drugs administered via this route. Especially visceral fat, located around abdominal organs, can act as a barrier or depot which in turn can cause a delayed onset of action [27,28]. Additionally, higher muscle mass may result in a larger volume of distribution which could necessitate a higher dose.

Increased levels of sMAC have been reported in many clinical settings, including autoimmune diseases, although interpretation is complicated by sample handling and assay variability [29]. For example, the impact of anesthetics on complement activation remains poorly defined. In humans, anesthetics have been shown to alter complement function, but whether this results in increased or reduced activity is unclear [30,31]. In a pilot study we also observed methodological variability, with lower sMAC levels detected in blood drawn from the vena cava (under anesthesia, after EMG and curare infusion), compared to blood sampled from the vena saphena without anesthesia (unpublished data). A clear difference was observed between PTMG anti-C2 treated animals and PTMG IC controls, suggesting systemic downstream complement inhibition. However, the reduction in sMAC was incomplete, likely reflecting sMAC serving as a general marker of systemic complement activation rather than a fully representative measure of MG pathology in this model. In comparison, local MAC deposition at NMJ is a well-established driver of tissue damage and clinical disease manifestations, making it a more relevant and meaningful readout of therapeutic efficacy in AChR-MG. Indeed, rats treated with anti-C2 antibodies hardly had MAC deposition at muscle endplates, indicating reduced complement activity. This was further supported by quantification of AChR by radioimmunoassay, which demonstrated no significant differences between anti-C2 treated PTMG and non-MG animals.

Despite growing interest in upstream complement inhibition, C2 remains an underexplored therapeutic target in MG, with no currently approved treatments. A C2-binding antibody, empasiprubart, is in clinical development for other indications, supporting its therapeutic potential for autoimmune diseases like MG [32] (NCT05225675). A proof-of-concept for C2 targeting therapies has been demonstrated using siRNA-mediated C2 knockdown in the EAMG mouse model [12]. However, this approach differs substantially from the antibody-based strategy used here. Monoclonal antibody therapy may offer advantages in clinical practice as they have generally a faster onset of action, predictable effects, and longer dosing intervals. Even more upstream investigational drugs such as narsoplimab (anti-MASP-2) and ANX005 (C1q inhibitor) have shown efficacy in preclinical studies of proteinuric nephropathy and motor axonal neuropathy [13,15] and taken together, these studies highlight C2 as a novel upstream complement inhibition target while emphasizing the need for pursuing therapeutic strategies directly targeting C2 for AChR-MG.

In conclusion, the results of this study indicate therapeutic potential of monoclonal antibodies targeting C2 in AChR-MG. Administration of anti-C2 antibodies to PTMG rats prevented characteristic development of MG symptoms and serum analyses confirmed inactivity of the targeted complement pathways. This targeted approach could offer an alternative to existing complement inhibitors, with potentially a broader application in complement-mediated disorders.

## 4 Methods

### 4.1 Experimental design and induction of PTMG

Female LEW/Crl rats (n=36) were purchased from Charles River Laboratories (Sulzfeld, Germany) and arrived at the Maastricht University animal facility at the age of 7-8 weeks of age. Rats were then housed in a reversed 12 h light-dark cycle, had *ad libitum* access to water and food and were fed a standard laboratory diet (R/M − H, Sniff Spezialdiäten GmbH, Soest, Germany). Animals had a 2-week acclimatization period upon arrival. In the subsequent week, animals were trained for 3 consecutive days for the muscle strength tests. Muscle strength and weight was recorded next (‘baseline’ measurements) and animals were then distributed into 4 experimental groups with a similar average weight (Table 1). At 10-11 weeks of age, animals received treatment consisting of I.P. injection of anti-rat C2 hIgG1 LALA (50 mg/kg) or the isotype control hIgG1 LALA (IC; 50 mg/kg) [4]. The anti-rat C2 hIgG1 LALA antibody binds specifically to the C2a side of rat C2 in a Ca^2+^-dependent manner. To reduce unwanted effector functions in rats, the antibody was formatted as hIgG1 with L234A/L235A (LALA) mutations. Single-dose pharmacokinetic and pharmacodynamic modeling revealed a reduction in free C2 concentrations ≥95% for a minimum duration of 48 h with a loading dose of 50 mg/kg.

**Table 1:** Experimental groups.

Group	Name	Animals (n)	Disease induction (dose)	Drug (dose)
1	PTMG IC	12	mAb35 (40 pmol/100 g BW)	isotype control (IC) (50 mg/kg)
2	PTMG anti-C2	12	mAb35 (40 pmol/100 g BW)	anti-rat C2 (50 mg/kg)
3	Non-MG IC	6	saline	isotype control (IC) (50 mg/kg)
4	Non-MG anti-C2	6	saline	anti-rat C2 (50 mg/kg)

Monoclonal antibody 35 hybridoma cells (mAb35; HB-8857) were purchased from ATCC and grown in suspension. Production, validation and quantification of mAb35 was performed as described [27]. Two hours after treatment with anti-C2 or isotype control, PTMG was induced by subcutaneously (S.C.) injecting 0.06 mg/kg mAb35 (40 pmol mAb35/100 g BW); non-MG (control) animals received an equal amount of saline. The injection volume was equally distributed between the nape and the flank using a cloth to restrain the animal. Injections were performed by a trained experimenter. Approximately 48 h after PTMG induction, terminal experiments and euthanasia were performed.

For practical reasons the experiment was performed with 6 batches of animals, with all experimental groups represented in each batch, starting the experiment subsequently, following the same timeline.

Animals underwent BW measurements, grip strength tests, disease scoring and welfare scoring of the following time points: 1) during baseline measurements, 2) on the day of (and prior to) PTMG induction, 3) 24 h after PTMG induction and 4) 48 h after disease induction. All assessments were performed blinded. Experiments were conducted with permission of the Committee of Animal Welfare, according to the Dutch laws, and in line with regulations of Maastricht University under the license AVD10700202216263. All investigators complied with the ARRIVE guidelines.

### 4.2 Disease assessment

Disease severity was assessed by general welfare scoring, BW, disease specific scoring and motor function (Supplementary Table S1). The frequency of assessment was increased to twice a day if symptoms reached a welfare score of 7. Disease-specific MG score was as follows: 0 = no disease symptoms before exercise; 1 = no clinical signs observed before testing, appearance of weakness after exercise due to fatigue; 2 = clinical signs present before testing, i.e. hunched posture, weak grip, or head down; 3 = severe clinical manifestation (no ability to grip, hindlimb paralysis, respiratory distress/dyspnea, immobility) and weight loss of 15% or more from the maximum BW recorded, leading to humane endpoint; and 4 = moribund [17]. Humane endpoint was defined by ≥15% loss of BW from the maximum recorded weight of that animal, not followed by recovery within a day and a combination of welfare score exceeding a score of 7 (Supplementary Table S1). Animals reaching humane endpoint were euthanized within 12 h of observation to prevent that animals would reach a disease score of 4.

### 4.3 Muscle strength

Rats’ muscle strength was assessed using a grip meter to evaluate forelimb strength and the hand climbing task for overall strength and fatigability. These two tasks were combined into a single assessment, representing a modification of the guidelines (Kusner et al., 2015). While held by the base of the tail, the animal was placed with forelimbs on the grip meter GS3 (Panlab Harvard Apparatus, Barcelona, Spain) and pulled backwards 5 consecutive times following the axis of the sensor. Peak-force generation was recorded automatically and the average of the 5 measurements was used for analysis. Subsequently, the animal was exercised with the hand climbing task. The experimenter held the rat by the base of the tail and allowed the rat to climb onto the dorsal side of the same hand while maintaining a 90° elbow angle. The experimenter’s arm and hand position remained fixed throughout the task to prevent interference with the animal’s movement. The exercise was repeated 5 times with 30 s resting intervals, lasting one attempt a maximum of 1 min. The number of successful climbs within the allotted time were noted. Animals that were able to climb all 5 times were categorized as successfully achieving the task. Correspondingly, not being able to climb up the hand led to categorization of failed.

### 4.4 Blood processing

Blood sampling from the saphenous vein took place at baseline (before disease induction) and 24 h post-disease induction. At the endpoint, blood was collected from the vena cava. Blood for serum collection was left for clotting for at least 30 min at room temperature (RT). The clot was removed by centrifuging 10 min at 18000g at RT. The resulting supernatant was collected and snap frozen on dry ice. All samples were stored at -80 °C until further use.

### 4.5 Electromyography, curare infusion and tissue collection

Rats received S.C. 0.05 mg/kg buprenorphine 1-4 h before the start of the terminal experiment. Animals were subsequently anesthetized by inhalation of 3 - 4% isoflurane and maintained under anesthesia at 1.5 - 3% isoflurane. EMG was performed as described [18,33]. In short, using monopolar needle electrodes the *nervus fibularis* was repeatedly stimulated every 30 s for 8 times supramaximal (0.2 ms per stimulus, 3 Hz) and response was recorded on the TA muscle. When animals did not show decrement (the amplitude between the first and fourth CMAP reduced by >10% for 3 consecutive recordings) within the first 10 min after inducing anesthesia, the animal underwent endotracheal intubation. The trachea was exposed and cut midway to introduce the endotracheal tube. Using a mechanical ventilator (VentElite, Harvard Apparatus) attached to the isoflurane dispenser, the animal was mechanically ventilated and kept under anesthesia. Subsequently, curare (T2379, Sigma-Aldrich) was infused I.P. using a syringe infusion pump (1 ml/h, 0.33 μg curare/minute, 20 μg/ml). During the procedure, the animal’s body temperature was kept between 36 and 38 °C using an infrared heating lamp (DISA, Copenhagen, Denmark) and a heating plate. The infusion time and corresponding curare dose at which a decrement was observed is indicative of the number of functional AChR at the NMJ. The isoflurane concentration was then increased to 5% and blood was extracted from the vena cava. Animals were euthanized by exsanguination under deep anesthesia, followed by cervical dislocation. TA muscles were collected snap frozen on dry ice for radioimmunoassay and frozen in isopentane for immunofluorescence. Tissue was stored at -80 °C until further use.

### 4.6 Free C2 ELISA

MaxiSorp microtiter plates were coated overnight with the anti-rat C2 antibody(Bracke et al., 2025) and blocked with SuperBlock T20 (PBS) (ThermoScientific, Cat N° 37516). After blocking, samples and calibration curve standards were pipetted into the ELISA plates and incubated on ice for 15 min. Serum samples were diluted in Low cross buffer (Candor, Cat N° 100500) diluted 1:2 with TBS (Tris Buffered Saline, ThermoFisher, Cat N°: BP24711) and supplemented with 2 mM CaCl_2_ (Sigma, Cat N°: 21115). As a calibration curve, dilutions of recombinant rat C2 (Argenx) in assay buffer were used. After incubation and washing, free C2 was detected with biotinylated human anti-rat C2 antibody 3H07 (Argenx). The epitope targeted by 3H07 is distinct from that recognized by the coated anti-rat C2 antibody. After incubation on ice, plates were developed with streptavidin-HRP and s(HS)TMB (SDT reagents, Cat N°: sTMB) 1:2 diluted in MilliQ. OD450/620 nm was measured with a spectrophotometer after the reaction was stopped with sulfuric acid.

### 4.7 Classical complement pathway activity ELISA (C3 deposition)

Microtiter wells (Greiner Bio-one, Cat N°: 675061) were coated with IgG (Bio-rad Laboratories, Cat N°: PMP01) and incubated overnight at 4 °C. After blocking with 1% w/v casein (Biorad, Cat N°: 1610783) and washing, wells were incubated for 5 min with rat serum samples diluted in veronal buffered saline at 37 °C. As controls, MgEGTA (inhibits classical and lectin complement pathways) or EDTA (inhibits all pathways) were added to the buffer. After washing, wells were incubated with biotinylated anti-rat C3 antibody (Abcam, Cat N°: ab17456), followed by streptavidin-HRP (Pharmingen, Cat N°: 554066). Afterwards, wells were washed and incubated with TMB solution. The substrate reaction was stopped with sulfuric acid and OD450/620 nm was measured with a spectrophotometer (Infinite M Nano, Tecan).

### 4.8 Soluble MAC ELISA

Soluble MAC in rat serum was measured using the Rat Terminal Complement Complex (TCC) kit (Hycult, Cat N° HK-106). The assay was performed according to the manufacturer’s recommendations, serum samples were diluted 300X in dilution buffer supplemented with 10 mM EDTA.

### 4.9 Radioimmunoassay for AChR content in TA

The AChR concentrations of isolated TA muscles were measured as described [18] with the following modifications. Three aliquots of 200 μl of muscle extract were prepared and incubated overnight at 4 °C with ^125^I-α-bungarotoxin (Revvity) and rat anti-torpedo AChR antibodies. Immune complexes were precipitated using 100 μl polyclonal goat anti-rat IgG (Eurogentec, Belgium). The measured radioactivity of ^125^I-α-bungarotoxin correlates to the amount of AChR present, which was normalized to tissue weight and non-MG values.

### 4.10 Qualitative immunofluorescence of NMJs

Qualitative immunofluorescence of NMJs was performed with modification to previously described methods [18,34]. Briefly, 10 μm cryosections of TA muscle, frozen in isopentane, were stained for AChR using α-bungarotoxin, and either for MAC or synaptic vesicle protein 2 (SV2). Dried cryosections were fixed with 1% formaldehyde (Sigma Aldrich #F8775) in PBS for 10 min at RT, followed by washing and blocking with 5% (v/v) normal goat serum (NGS) in PBS for 30 min at RT. Slides were incubated overnight at 4 °C with either mouse anti-C5b-9 (1:50, 4 μg/ml, Santa Cruz Biotechnologies #sc-66190) or mouse anti-SV2 (1:2000, 1.55 μg/ml, DSHB #AB_2315387). After washing, slides were incubated for 1 h at RT with biotinylated goat anti-mouse IgG (1:400, 2.5 μg/ml, Jackson Immunoresearch #115-065-166). This was followed by incubation 1 h at RT with streptavidin Alexa Fluor 594 (1:500, 4 μg/ml, Invitrogen #S-32356), α-bungarotoxin Alexa Fluor 647 (1:300, 3.33 μg/ml, Invitrogen #B35450) and DAPI (1:800). All antibodies were diluted in 2% (w/v) NGS in PBS. Washing steps consisted of two 5 min incubations in 0.05% (v/v) TritonX-100 in PBS, followed by a final 5 min incubation in PBS, all performed at RT. Slides were mounted using 80% (v/v) glycerol in PBS. Images were acquired with a fluorescence Olympus BX51WI microscope (Olympus, Hamburg, Germany), connected to a Hamamatsu EM-CCD C9100 camera (Hamamatsu Photonics K. K., Hamamatsu City, Japan), controlled with Micromanager software (version 2.0.3). Intensities were adjusted using ImageJ version 1.48a (NIH) as previously described [25].

### 4.11 Statistical methods

GraphPad Prism 10 was used to perform statistical analyses. Comparisons between the BW, grip strength, complement protein assays, curare dose and muscle AChR content of experimental groups were performed using one way ANOVA. For the hand climbing test a Fisher’s exact test was performed. A Bonferroni test was applied to correct for multiple comparisons in both methods. For statistical analyses of clinical scores and C3 expression a Kruskal-Wallis test with Dunn’s test was performed. Statistical significance in the validation study was determined using a *t*-test. A two-sided probability value of p≤0.05 was considered significant.

## Funding

This study was funded by argenx.

## CRediT authorship contribution statement

**Britt Arets:** Formal analysis, Investigation, Validation, Writing – original draft. **Siyang Peng:** Data curation, Investigation, Validation, Writing – review & editing. **Laura Bracke:** Data curation, Formal analysis, Investigation, Methodology, Writing – review & editing. **Karen De Winter:** Data curation, Formal analysis, Investigation, Validation, Writing – review & editing. **Jolien Delaere:** Data curation, Formal analysis, Investigation, Validation, Writing – review & editing. **Yasmine Driege:** Data curation, Formal analysis, Investigation, Validation, Writing – review & editing. **Tim Delahaye:** Data curation, Formal analysis, Investigation, Methodology, Validation, Writing – review & editing. **Mahan Moshir:** Conceptualization, Data curation, Formal analysis, Funding acquisition, Validation, Writing – review & editing. **Inge Van de Walle:** Funding acquisition, Resources, Writing – review & editing. **Peter Ulrichts:** Conceptualization, Funding acquisition, Investigation, Writing – review & editing. **Marina Mané-Damas:** Conceptualization, Data curation, Formal analysis, Investigation, Methodology, Supervision, Writing – review & editing. **Mario Losen:** Conceptualization, Data curation, Formal analysis, Investigation, Supervision, Validation, Writing – review & editing. **Pilar Martinez-Martinez:** Conceptualization, Investigation, Methodology, Project administration, Resources, Supervision, Writing – review & editing.

## Declaration of Competing Interest

The authors declare the following financial interests/personal relationships which may be considered as potential competing interests: Pilar Martinez-Martinez reports financial support was provided by Maastricht University. LB, KDW, JD, YD, TD, MM, IVW and PU are current employees of argenx. If there are other authors, they declare that they have no known competing financial interests or personal relationships that could have appeared to influence the work reported in this paper.

## Data Availability

Data will be made available on request.
