# Supplementary material for: Anti-C2–mediated complement inhibition prevents disease in a rat passive transfer model of myasthenia gravis
Source: J Transl Autoimmun. 2026 Jul 16;13:100388. doi: 10.1016/j.jtauto.2026.100388 (PMC13445179; doi:10.1016/j.jtauto.2026.100388)
Supplement: Supplementary file 1 [file mmc1.docx]

**Supplementary**

**Supplementary Table S1.** MG-related welfare score sheet

| **Description** | | | | | |
| --- | --- | --- | --- | --- | --- |
| **General impression** | | | | | |
|  | **0** | **1** | **2** | **3** | **HEP*** |
| **Posture** | Normal |  | Hunched |  | Flaccid paralysis |
| **Movement**  **Activity**  **Awareness** | Normal | Slightly reduced activity | Lack of awareness  Markedly reduced and/or uncoordinated activity  Reluctant to move, tremor Resting neck after some activity | Significant mobility problems: Incapacity to keep the neck straight in resting position, persisting immobility<24h | Flaccid paralysis |
| **Coat**  **Skin condition** | Normal | Rough  Slightly unkempt | Slight pilo-erection  Rough fur  Lack of grooming  Slightly dry skin | Marked pili-erection |  |
| **Breathing pattern**  **(before activity)** | Normal | Rapid/ Accelerated breathing (tachypnoea) |  | Audible breathing not related to MG (excl. grunting), laboured breathing (hyperpnoea) | Laboured breathing (dyspnoea) |
| **Body weight loss %** | 0-5% | >5-10% | >10-15% | >15%-20% | >20% |
| **Hydration** | Normal | Skin less elastic  (slightly delayed) | Skin tents  (delayed) | Skin tents  (very delayed) | Skin tents  (very delayed), eyes sunken |
|  | **0** | **1** | **2** | **3** | **HEP*** |
| **Motor function; Rack grabbing test** | no weakness | weakness after completing activity | weakness and fatigability during activity;  incapable to finish the task | severe weakness; incapability to start the task |  |
| **Motor function; Hand climbing test** | no weakness | weakness after completing activity | weakness and fatigability during activity;  incapable to finish the task | severe weakness; incapability to start the task |  |

*Humane endpoint (HEP) implemented; regardless of other clinical signs

The welfare score sheet is a guideline for assessment. In case for the total score the corresponding conditions are not met, consult the research team to discuss the following steps.

| **Total Score** | **Conditions** | **Action if condition is met** |
| --- | --- | --- |
| 1 or 2 | - | No action needed |
| 3 | Either one score of 2 or three of 1 | Review monitoring frequency |
| 4 or 5 | At least one score of 2  Or one score 3 | Consider supplementary fluids, care or analgesia |
| 6 or 7 | At least two scores of 2  Or one score of 3 | Review progress, consult veterinarian |
| 8 | Combination of two scores of 3 and one score of 2 | HEP |

**Supplementary Figure S1**. Relative grip strength at endpoint compared to baseline. Loss of grip strength at endpoint (48 hours) was determined by dividing the difference in grip strength at 48 hours compared to baseline, by the baseline strength.
